# Evidence-based geriatric knowledge among healthcare providers in Vietnam: adaptation, validation, and pilot of the knowledge about older patients quiz

**DOI:** 10.1186/s12877-023-03958-3

**Published:** 2023-05-12

**Authors:** Oluwarantimi Adetunji, David Bishai, Cuong Viet Pham, Janiece Taylor, Ngan Tran Thi, Zainab Khan, Abdulgafoor M. Bachani

**Affiliations:** 1 Johns Hopkins International Injury Research Unit, Health Systems Program, Department of International Health, Johns Hopkins Bloomberg School of Public Health, Baltimore, MD USA; 2 Center for Injury Policy and Prevention Research (CIPPR), Hanoi University of Public Health, Hanoi, Vietnam; 3 Johns Hopkins School of Nursing, Baltimore, MD USA

**Keywords:** Geriatric care, Aging, Nurses, Knowledge, Healthcare quality, Cross-cultural adaptation, Vietnam, Healthcare providers, Older adults, Elderly

## Abstract

**Background:**

Vietnam’s aging population is growing rapidly, but its health workforce’s capacity to provide quality geriatric care is not clearly understood. We aimed to provide a cross-culturally relevant and validated instrument to assess evidence-based geriatric knowledge among healthcare providers in Vietnam.

**Methods:**

We translated the Knowledge about Older Patients Quiz from English to Vietnamese using cross-cultural adaptation methods. We validated the translated version by evaluating its relevance to the Vietnamese context, as well as its semantic and technical equivalence. We fielded the translated instrument on a pilot sample of healthcare providers in Hanoi, Vietnam.

**Results:**

The Vietnamese Knowledge about Older Patients Quiz (VKOP-Q) had excellent content validity (S-CVI/Ave) and translation equivalence (TS-CVI/Ave) of 0.94 and 0.92, respectively. The average VKOP-Q score was 54.2% (95% CI: 52.5—55.8) and ranged from 33.3 to 73.3% among 110 healthcare providers in the pilot study. Healthcare providers in the pilot study had low scores on questions related to the physiopathology of geriatric conditions, communication techniques with sensory impaired older adults, and differentiating age related changes from abnormal changes or symptoms.

**Conclusions:**

The VKOP-Q is a validated instrument to assess geriatric knowledge among healthcare providers in Vietnam. The level of geriatric knowledge among healthcare providers in the pilot study was unsatisfactory, which supports the need for further assessment of geriatric knowledge among a nationally representative sample of healthcare providers.

**Supplementary Information:**

The online version contains supplementary material available at 10.1186/s12877-023-03958-3.

## Background

The geriatric knowledge of healthcare providers is associated with the quality of care provided to older people [1, 2]. Geriatric knowledge is the understanding of aging and its implications for appropriate health care and treatment of older people to maximize their functional capacity [3]. Geriatric knowledge is crucial for healthy aging because inappropriate geriatric care for older adults can accelerate functional decline, including iatrogenic disability and adverse health outcomes [4, 5]. Prior studies have linked poor geriatric knowledge among healthcare providers to an increased risk for falls, hyperglycemia, hypoglycemia, abuse, functional decline, and mortality among older adults [6–8].

The aging population in Vietnam is growing rapidly. The national aging survey revealed that two-thirds of older adults self-rated their health as below normal, with chronic disease as the most prevalent cause of illness among them [9]. While social protection for older adults has been extensively studied in Vietnam, few researchers have examined the health workforce’s capacity to provide quality geriatric care [10–13]. An assessment revealed that some of the health facilities with geriatric departments, especially in rural areas, were staffed by healthcare providers with no geriatric training [14]. In response to its aging population needs, Vietnam’s Ministry of Health (MOH) introduced a guideline to care for older adults and outlined a national action plan to increase the capacity of the health system to provide quality geriatric care. However, these initiatives have not been evaluated for their effectiveness in improving geriatric knowledge among healthcare providers. The field of knowledge translation research has documented that the adoption and implementation of new guidelines into practice is slow and may not happen at all [15]. Therefore, this study aimed to provide an appropriate instrument to monitor the impact of interventions at the individual, organizational, and national levels on the geriatric knowledge among healthcare providers.

The goal of this study was to provide an instrument to assess evidence-based geriatric knowledge among healthcare providers in Hanoi, Vietnam. We translated, validated the cross-cultural relevance, and piloted the Knowledge about Older Patients Quiz (KOP-Q) in Vietnam. In addition, we conducted a pilot study to identify potential gaps in the geriatric knowledge among the healthcare workforce, specifically nurses and physicians.

## Methods

KOP-Q is a validated and psychometrically sound instrument originally designed to assess gaps in the geriatric knowledge among nurses in the Netherlands. It contains 30 dichotomous (true or false) statements to measure the knowledge of healthcare providers about the appropriate care for hospitalized older adults. It measures the healthcare providers’ certainty or confidence in their responses [16]. The Consensus-based Standards for the selection of health Measurement Instruments (COSMIN) were applied in the development of the KOP-Q [17, 18]. It demonstrated good item characteristics, reliability for the knowledge items (Kuder-Richardson Formula 20 = 0.70), and a good scale content validity (S-CVI/average = 0.91). The certainty items had excellent reliability (Cronbach’s alpha = 0.94) [16]. KOP-Q was cross-culturally translated from Dutch to English and validated for use in the United States [19].

### Translation and cross-cultural validation of the KOP-Q to the vietnamese context

Table 1 illustrates the translation process that was based on cross-cultural adaptation methods to assess cultural relevance, as well as the semantic and technical equivalence of the instrument [20–22]. The study used the forward-backward method to translate the KOP-Q instrument. The forward-backward approach is a popular method for instrument validation for cross-cultural research [23]. A bilingual person, who was a native Vietnamese speaker, translated the instrument from English to Vietnamese. This translated version was reviewed and revised by a panel of 5 bilingual researchers. Then another bilingual translator, who did not see the original English version of the instrument, translated the Vietnamese version to English. The English back-translation was compared to the original version of the instrument to detect alterations in the meaning. Geriatric expert researchers and practitioners discussed discrepancies to reach a consensus, which is the recommended process for the cross-cultural adaptation of instruments [24].

An expert review panel of bilingual geriatric researchers and practitioners evaluated the cross-cultural relevance and translation equivalence of the KOP-Q Vietnamese translation. Experts rated each question on a scale from 1 to 4 (not relevant, somewhat relevant, very relevant or highly relevant) using an online data collection tool. Then they were asked to compare the translation to the original English text and rated the equivalence of the translation as either yes or no. We summarized the demographics of the expert panel and calculated Content Validity Indexing (CVI) scores to measure the degree of agreement between raters to predict potentially problematic instrument items [25].

A total of seven bilingual experts rated the technical equivalence and cross-cultural relevance of the Vietnamese Knowledge about Older Patients Quiz (VKOP-Q) via an online tool. The sample size of seven was sufficient to conduct the cross-cultural and translation equivalency analyses [26]. Out of the seven experts, three were public health researchers, three were physicians, and one was a nurse. All of them had at least one postgraduate degree and the average years of clinical experience was 12 years. Five experts with clinical experience in the past two years reported providing health care to older adults. Six experts reported prior experience conducting research, four of whom had expertise in geriatrics. The average years of research experience was 11.8 years.

CVI scores were calculated at both the item (I-CVI) and scale (S-CVI) levels to assess the relevance of the KOP-Q to geriatric care in Vietnam [26]. The I-CVI is calculated by summing the number of raters scoring an item as 3 or 4 (very or highly relevant) divided by the total number of expert raters. The I-CVI, between 0 and 1, measures the proportion of agreement on the relevance of each item [27]. The S-CVI measures the proportion of the instrument judged relevant. S-CVI may be calculated either as the averages of all the item level CVIs (S-CVI/Ave) or the universal agreement among the experts (S-CVI/UA), which is considered stringent [27]. For the S-CVA/UA, the total number of items with I-CVI equal to 1, that is rated relevant by all experts, was divided by the total number of questions in the KOP-Q [26]. In order to measure the translation equivalence, we used TI-CVI and TS-CVI scores developed by Squire and colleagues that was based on the CVI approach [20]. The content relevance and translation equivalence scores were calculated separately.

Since there is some chance agreement among experts, the I-CVI score was also translated into a modified kappa statistic calculation using this formula:

$${K}_{m}= \frac{\text{I}-\text{C}\text{V}\text{I} -{P}_{c}}{1-{P}_{c}} ,$$

where *K*_m_ is the modified kappa and *P*_c_ is the probability of chance agreement. The probability of chance agreement was calculated as:

$${P}_{c}= \frac{\text{N}!}{A!-\left(N-A\right)!}*{0.5}^{N},$$

where N is the number of experts and A is the number of experts who agree that the item is relevant.

**Table 1:** Illustration of Systematic Cross-Cultural Translation and Evaluation Process

Translation Guide [20]	Identified potentially problematic questions, phrases or terms that may be difficult to translate conceptually. Wrote equivalent terms or phrases through free-listing techniques. Provided translation guide to translators.
Forward Translation [21, 28]	2 translations of survey from English to Vietnamese. Bilingual native Vietnamese speaker, who was familiar with geriatric knowledge and patient-centered care. Translators documented challenging phrases or uncertainties, as well as rationale for word choices.
Back Translation [21, 28]	Bilingual native English speaker, who was unfamiliar with geriatric knowledge and patient-centered care. Translators documented challenging phrases or uncertainties, as well as rationale for word choices. 3 researchers, compared back translation to original English survey to resolve discrepancies by consensus.
Expert Panel Review using Content Validity Indexing [20]	Utilized Content Validity Indexing (CVI) method to evaluate equivalence and relevance of survey questions. 7 bilingual practicing nurses, physicians, and researchers with content and/or methodological expertise. Rated content, construct, and conceptual relevance of questions to nursing practice in Vietnam. Evaluated semantic and technical equivalence of translation. Provided comments about responses.
Finalize Translation [20]	Finalized translation based on CVI feedback. Documented all problem questions for reference during data collection and analysis.
Pre-test [21, 28]	Trained data collectors. Conducted test run of instrument in the field. Documented all problem questions for reference.
Pilot [20]	Collected data at health facilities.

The modified kappa statistic focuses on the agreement of relevance and a value above 0.74 is excellent, 0.60 to 0.74 is good, and 0.4 and 0.59 is fair [20, 26 ,29]. On the other hand, an I-CVI score ≥ 0.78 is considered to have excellent content validity [26]. Nevertheless, the values of I-CVI and modified kappa converge with an increase in sample size (N ≥ 10) [26]. An instrument with an S-CVI/Ave score ≥ 0.90 is judged to have excellent content validity. The minimum acceptable values for I-CVI and S-CVI/Ave are 0.50 and 0.80 respectively [26]. The results from the S-CVI/Ave, modified kappa, and I-CVI were used as the evaluation criteria [26]. Microsoft Excel 2016 was used for all CVI calculations in the study.

#### Piloting the VKOP-Q in Vietnam

**Fig. 1:**
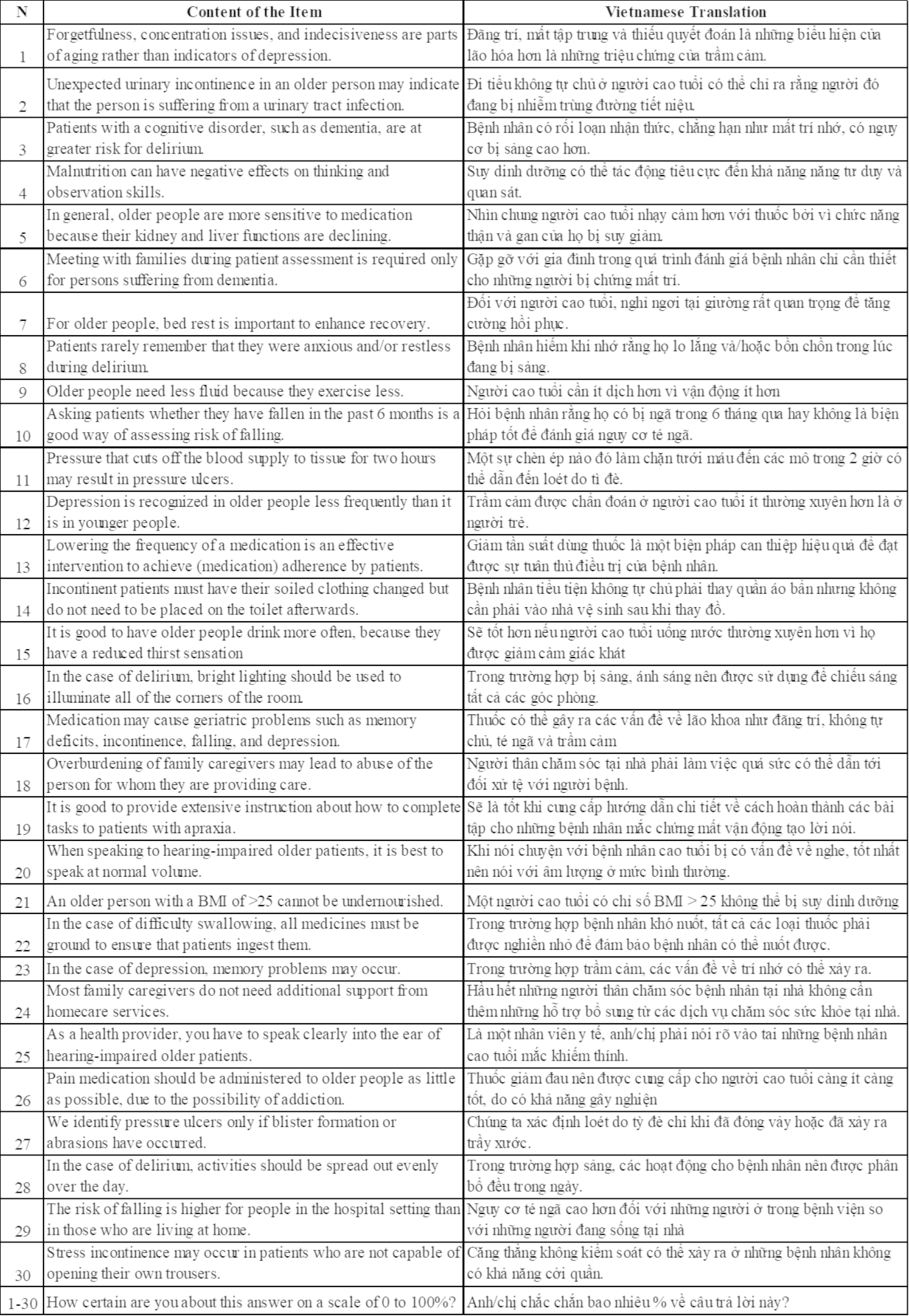
English and Vietnamese versions of Knowledge about Older Patients-Quiz

The study was reviewed and approved by the Institutional Review Boards at Johns Hopkins Bloomberg School of Public Health and Hanoi University of Public Health. Prior approvals were obtained from the administrative leaders of each health facility before data collection. Data collectors participated in a two-day training, including pre-testing at a health facility. The finalized VKOP-Q incorporated feedback from the expert panel and pre-test in the field. The VKOP-Q is shown in Fig. 1.

The VKOP-Q was administered to nurses and physicians in 30 facilities in Hanoi between March and April 2019. At least 110 participants were recruited to allow attrition from missing data. The study sample size was calculated with statistical power analysis using the effect size, probability of not having a type II error (power), and the probability of committing a type I error (α) [30]. Power and alpha were set at 0.80 and 0.05, respectively.

Prior studies suggested a meaningful difference of 10% between groups would be adequate and led us to expect a standard deviation of 10–15% of the geriatric knowledge scores within each group [31–33]. Similarly, the effect sizes for correlations between the geriatric knowledge score and the independent variables were determined to be medium (i.e. with R^2^ of 0.13) based on prior studies [19, 31, 32, 34]. Cohen [30] defined small, medium, and large effect sizes for multiple regression were defined as R^2^ of 0.02, 0.13, and 0.26 respectively. Thus, a minimum sample size of 100 was required to avoid type II error with a medium effect size.

Convenience sampling strategy was used to select 30 health facilities from two urban districts and three suburban districts. Quota sampling method was used so that the sample was proportional to the health facility size. Maximum values of 2, 10, and 20 participants per health facility was assigned at the commune, district/provincial, and central levels, respectively. Average values of 5, 76, and 135 healthcare providers was eligible per each health facility at the commune, district/provincial, and central levels, respectively [35, 36].

At the health facilities, clinical supervisors facilitated the recruitment of healthcare providers up to the assigned quota. Healthcare providers were invited to participate voluntarily in the study. In order to minimize the disruption of health services, healthcare providers participated in the study when it was convenient for their schedule. A stipend of VND100,000 (~$5) was provided to each respondent.

The instrument data were entered into a form on KoBo Toolbox, a secure web-based application for data collection and management [37]. Data entry was confirmed by two researchers. Data were exported to Stata 15 software for analysis. Data were cleaned systematically, and a series of logic checks were performed on the demographic variables. The data collected from healthcare providers were coded according to the instructions provided by the author, which is based on classical test theory (CTT) that sums up correctly answered questions [16, 31]. Knowledge questions with “don’t know” were counted as having wrong answers. The knowledge score was transformed into a percentage by dividing the score by 30 and multiplying it by 100. Satisfactory knowledge score was at least 24 out of 30 points, which is equivalent to 80% [31]. Each respondent had one knowledge score and one certainty score.

Descriptive statistics were calculated for the characteristics of healthcare providers that participated in the study, disaggregated by occupation. Multiple linear regression (MLR) model was specified to estimate the association between the dependent variable and the characteristics of healthcare providers, adjusting for confounding factors. The dependent variable was the aggregate geriatric knowledge scores out of 30 points. The MLR model was constructed applying forward selection. A difference of statistical significance was established at *p*-values less than 0.05. Akaike information criterion (AIC), Bayesian information criterion (BIC), and R^2^ values were used for model selection.

In order to identify gaps in geriatric knowledge, the proportion of correct responses was examined for each VKOP-Q item. The statistical significance of differences in the proportion of correct response among groups for each item was examined by Fisher’s exact test for categorical variables. The themes of the higher and lower scored questions of the VKOP-Q were identified and described for the full sample and by groups. Differences in group means of the certainty construct were also examined with ANOVA for each VKOP-Q item.

## Results

### Content and translation validation

Table 2 shows the complete results of the CVI indices. *K*m values were identical to the CVI indices. On the item level, 29 (97%) out of 30 questions had excellent validity ratings and one item had a good validity rating. None of the I-CVI was below 0.50, which is the criteria for rejection. The S-CVI/Ave was 0.94, which indicated excellent overall content validity and was comparable to the S-CVI/Ave score of 0.91 for the original scale. Similarly, 93% (28) of the items had TI-CVI scores ≥ 0.78, which means the translation equivalence was rated excellent. The remaining two questions had good translation equivalence. The TS-CVI/Ave was 0.92, which means that the overall translation equivalence of the instrument was rated excellent.

### Geriatric knowledge among healthcare providers

Participants were recruited from 30 health facilities at all four levels across 5 districts in Hanoi. In total, 112 healthcare providers were interviewed between March and April 2019. Table 3 summarizes the descriptive statistics, disaggregated by occupation, for the sample of healthcare providers in the study.

**Table 2:** Content and Translation Validity of the KOP-Q to Vietnamese

N	Content of the item	Content Relevance				Translation Equivalence			
		Expert (N)	Rating ≥ 3(N)	I-CVI	Evaluation^*^	Expert (N)	Rating of 1 (N)	TI-CVI	Evaluation^*^
1	Forgetfulness, concentration issues, and indecisiveness are parts of aging rather than indicators of depression.	7	7	1.00	Excellent	7	7	1.00	Excellent
2	Unexpected urinary incontinence in an older person may indicate that the person is suffering from a urinary tract infection.	7	7	1.00	Excellent	7	7	1.00	Excellent
3	Patients with a cognitive disorder, such as dementia, are at greater risk for delirium.	7	6	0.86	Excellent	7	7	1.00	Excellent
4	Malnutrition can have negative effects on thinking and observation skills.	7	7	1.00	Excellent	7	7	1.00	Excellent
5	In general, older people are more sensitive to medication because their kidney and liver functions are declining.	7	7	1.00	Excellent	7	7	1.00	Excellent
6	Meeting with families during patient assessment is required only for persons suffering from dementia.	7	6	0.86	Excellent	7	5	0.71	Good
7	For older people, bed rest is important to enhance recovery.	7	7	1.00	Excellent	7	6	0.86	Excellent
8	Patients rarely remember that they were anxious and/or restless during delirium.	7	5	0.71	Good	7	6	0.86	Excellent
9	Older people need less fluid because they exercise less.	7	7	1.00	Excellent	7	6	0.86	Excellent
10	Asking patients whether they have fallen in the past 6 months is a good way of assessing the risk of falling.	7	7	1.00	Excellent	7	6	0.86	Excellent
11	Pressure that cuts off the blood supply to tissue for two hours may result in pressure ulcers.	7	6	0.86	Excellent	7	7	1.00	Excellent
12	Depression is recognized in older people less frequently than it is in younger people.	7	6	0.86	Excellent	7	6	0.86	Excellent
13	Lowering the frequency of a medication is an effective intervention to achieve (medication) adherence by patients.	7	7	1.00	Excellent	7	7	1.00	Excellent
14	Incontinent patients must have their soiled clothing changed but do not need to be placed on the toilet afterwards.	7	7	1.00	Excellent	7	6	0.86	Excellent
15	It is good to have older people drink more often, because they have a reduced thirst sensation	7	6	0.86	Excellent	7	7	1.00	Excellent
16	In the case of delirium, bright lighting should be used to illuminate all of the corners of the room.	7	6	0.86	Excellent	7	6	0.86	Excellent
17	Medication may cause geriatric problems such as memory deficits, incontinence, falling, and depression.	7	6	0.86	Excellent	7	7	1.00	Excellent
18	Overburdening of family caregivers may lead to abuse of the person for whom they are providing care.	7	6	0.86	Excellent	7	6	0.86	Excellent
19	It is good to provide extensive instruction about how to complete tasks to patients with apraxia.	7	7	1.00	Excellent	7	7	1.00	Excellent
20	When speaking to hearing-impaired older patients, it is best to speak at normal volume.	7	7	1.00	Excellent	7	6	0.86	Excellent
21	An older person with a BMI of > 25 cannot be undernourished.	7	7	1.00	Excellent	7	6	0.86	Excellent
22	In the case of difficulty swallowing, all medicines must be ground to ensure that patients ingest them.	7	7	1.00	Excellent	7	7	1.00	Excellent
23	In the case of depression, memory problems may occur.	7	7	1.00	Excellent	7	7	1.00	Excellent
24	Most family caregivers do not need additional support from homecare services.	7	7	1.00	Excellent	7	7	1.00	Excellent
25	As a health provider, you have to speak clearly into the ear of hearing-impaired older patients.	7	7	1.00	Excellent	7	7	1.00	Excellent
26	Pain medication should be administered to older people as little as possible, due to the possibility of addiction.	7	7	1.00	Excellent	7	7	1.00	Excellent
27	We identify pressure ulcers only if blister formation or abrasions have occurred.	7	6	0.86	Excellent	7	6	0.86	Excellent
28	In the case of delirium, activities should be spread out evenly over the day.	7	7	1.00	Excellent	7	6	0.86	Excellent
29	The risk of falling is higher for people in the hospital setting than in those who are living at home.	7	7	1.00	Excellent	7	6	0.86	Excellent
30	Stress incontinence may occur in patients who are not capable of opening their own trousers.	7	6	0.86	Excellent	7	5	0.71	Good
		S-CVI/Ave		0.94		TS-CVI/Ave		0.92	
		S-CVI/UA		0.63		TS-CVI/UA		0.5	

^*^ Evaluation criteria for the level of validity: excellent validity = I-CVI ≥ 0.78 and *K*_m_ >0.74; good validity I-CVI < 0.78 and ≥ 0.60 and *K*_m_ ≤ 0.74 [38]

**Table 3:** Descriptive Statistics

Variables	Total (N = 112)		Nurses (N = 71)	Physicians (N = 41)
	N	%	%	%
Occupation				
Nurses	71	63.4	100	-
Physicians	41	37.6	-	100
Females	83	74.1	83.1	58.5
Any postgraduate education	31	27.7	23.9	34.1
Any prior formal geriatric training	71	63.4	64.8	61.0
Years of experience, *mean (SD)*	112	12.6 (0.8)	12.6 (1.0)	12.6 (1.4)
Number of patients per day, *mean (SD)*	110	14.9 (1.2)	12.8 (1.3)	18.6 (2.3)
Number of older adult patients per day, *mean (SD)*	110	10 (0.9)	8.4 (0.9)	12.6 (1.8)
Urban location	53	47.3	49.3	58.5
Districts				
Cau Giay	18	16.1	14.1	19.5
Dong Anh	35	31.3	38.0	19.5
Dong Da	11	9.8	7.0	14.6
Dan Phuong	40	35.7	35.2	36.6
Long Bien	8	7.1	5.6	9.8
Health facility levels				
Commune	43	38.4	45.1	26.8
District or Provincial	29	25.9	19.7	36.6
Central	40	35.7	35.2	36.6
Departments				
Geriatric	19	17.0	18.3	14.6
Primary	48	42.9	49.3	31.7
Internal	26	23.2	22.5	24.4
Cardiology	16	14.3	12.7	17.1

The majority were nurses (63.4%) or female (74.1%). Most nurses were either 2-year secondary (40.1%) or 3-year college (35.2%) nurses, while most physicians were general doctors (63%). Almost two-thirds (63.4%) reported prior formal geriatric training. Geriatric training was reported by 60.1%, 24.1%, and 95.0% of respondents at communes, district, and central health facilities respectively. Postgraduate education was reported by 4.7%, 13.8%, and 62.5% of respondents at communes, district, and central health facilities respectively. The average years of work experience was 12.6 years (95% CI: 11.0—14.2). Participants reported providing services to an average of 10 (95% CI: 8.3—11.7) older adult patients per day and 15 (95% CI: 12.6—17.3) total patients per day.

Data quality was good with only 11 participants selecting a “don’t know” response for the knowledge questions. The mean knowledge score was 16.3 (95% CI: 15.7—16.8) of a possible 30, which indicates a low level of geriatric knowledge among respondents. On a transformed 100-point scale, the average score was 54.2% (95% CI: 52.5—55.8) and the scores ranged from 33.3 to 73.3%. None of the respondents had a perfect score, none of the statements were answered correctly by all respondents, and none of the respondents had a satisfactory knowledge score.

**Fig. 2:**
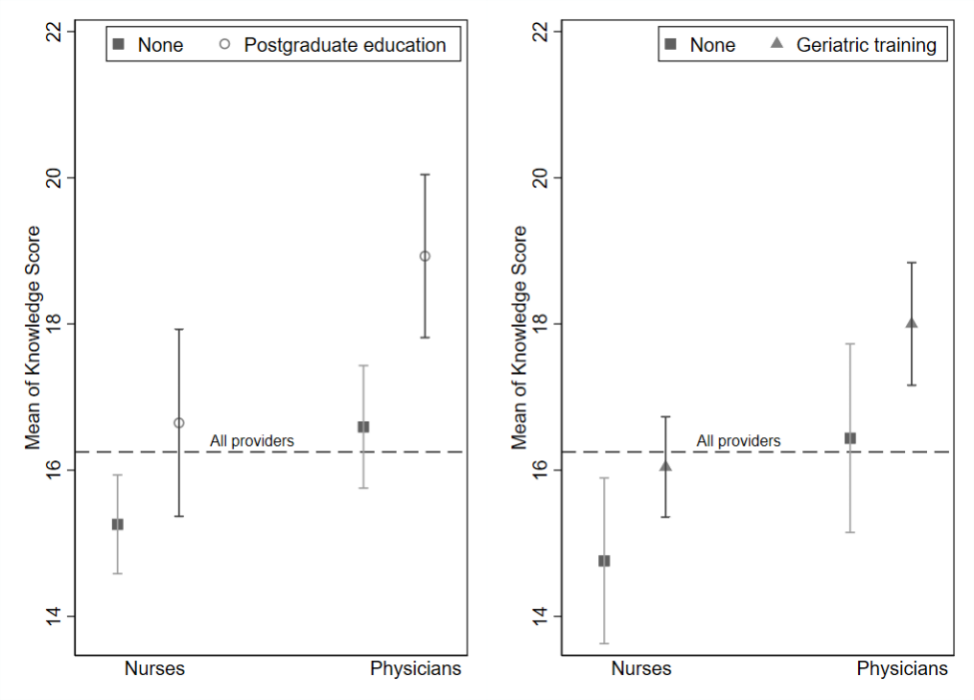
Knowledge scores by occupation, postgraduate education and geriatric training

Of the 30 statements, 12 questions were answered incorrectly by over 50% of respondents. The mean knowledge score was significantly higher among physicians, healthcare providers with geriatric training, and healthcare providers with postgraduate education (Fig. 2). Healthcare providers at central level facilities scored higher than lower-level facilities. The mean certainty score was 82.2 (95% CI: 80.6—83.9) and healthcare providers with prior geriatric training had significantly higher geriatric knowledge than their counterparts.

The Shapiro-Wilk test showed that both the geriatric knowledge (*p* = 1.0) and certainty scores (*p* = 0.57) were normally distributed. Two respondents were missing values for the average number of older patients per day and were excluded from the regression analysis. Results from the regression models for geriatric knowledge scores were presented in Table 4. In the MLR model, physicians had higher geriatric knowledge than nurses. Postgraduate education was positively associated with geriatric knowledge. The self-report of prior geriatric training was no longer associated with higher geriatric knowledge.

**Table 4:** Regression Coefficients for Geriatric Knowledge^i^

Variables	Unadjusted Coefficient	95% CI	Adjusted Coefficient	95% CI
Sex				
Female	1	-	1	-
Male	0.78	-0.37–1.92	0.28	-0.83–1.39
**Occupation**				
Nurses	1	-	1	
Physicians	1.80***	0.80–2.79	1.40**	0.34–2.45
**Any postgraduate education**				
No	1	-	1	
Yes	1.97***	0.91–3.04	1.33**	0.19–2.47
**Any prior geriatric training**				
No	1	-	1	
Yes	1.32**	0.30–2.34	0.93*	-0.11–1.96
**Years of experience**				
0–9 years	1	-	1	-
10 + years	-0.71	-1.72–0.29	-0.62	-1.61–0.37
**Number of older adult patients daily**	0.07**	0.01–0.12	0.04	-0.01–0.10
**Certainty score**	0.07**	0.01–0.13	0.03	-0.02–0.09
Constant			11.87***	7.09–16.65
Observations			110	
R-squared			0.28	

^i^ Model was adjusted for the interviewer effect, which was not significant

*** p < 0.01, ** p < 0.05, * p < 0.1

### Gaps in the geriatric knowledge of healthcare providers

Since there were differences in the geriatric knowledge scores across groups, we analyzed the number and proportion of correct responses and certainty scores for each VKOP-Q item, disaggregated by occupation and highest education level of the respondents. Secondary nurses had the lowest knowledge score with an average of 49.0% (95% CI: 46.0—52.0), while physicians with postgraduate education had the highest knowledge score with an average of 63.1% (95% CI: 59.3—66.9) correct responses. Healthcare providers at lower-level facilities (commune health centers, district and provincial facilities) had lower 51.3% (95% CI: 49.5—53.2) geriatric knowledge than the average score of 59.3% (95% CI: 56.6—61.9) at central facilities.

VKOP-Q items with a high proportion of correct responses were related to the knowledge of appropriate family interventions for geriatric care [Appendix]. Of the 30 statements, 12 and 6 questions were answered incorrectly by over 50% and 80% of respondents, respectively. However, respondents reported high certainty for those questions. VKOP-Q items with a low proportion of correct responses were related to the pathophysiology of geriatric conditions (depression, delirium, dementia, incontinence, pressure ulcers, polypharmacy, nutrition, and falling), communicating techniques with hearing or speaking impaired older patients, and differentiating between vulnerable versus older patients. VKOP-Q items with statistically significant differences among the groups of healthcare providers were related to the knowledge of geriatric conditions, with lower scores among nurses and lower formal education.

## Discussion

This study is the first validation of the VKOP-Q among healthcare providers in Vietnam. VKOP-Q measures evidenced-based geriatric knowledge construct solely, instead of opinions or attitudes. VKOP-Q assesses a construct on the certainty about geriatric knowledge, which is a measure of the ability of respondents to reflect on their own knowledge [32]. The translation of the KOP-Q to Vietnamese followed international standards for cross-cultural adaptation of surveys in health services research to reduce the threats to data validity and improve instrument reliability [20, 23, 24, 28]. The S-CVI/Ave and TS-CVI/Ave scores for the VKOP-Q demonstrated excellent content validity and translation equivalence [26].

The validated VKOP-Q is relevant because of the demographic transition to an aging population in Vietnam [39, 40]. In this study, healthcare providers reported that the majority (67%) of their current daily patient volume were older adults. The demand for health care from older adults is expected to increase with the rapidly aging population and underscores the need for health workers to be knowledgeable about geriatric care [41].

 This study provides an appropriate instrument for policymakers and administrators to assess the baseline and effectiveness of interventions targeted at improving evidence-based geriatric knowledge among healthcare providers.

The VKOP-Q average score suggested an unsatisfactory level of geriatric knowledge among the convenience sample of healthcare providers in the pilot study. The range of knowledge scores was comparable to the reported geriatric knowledge scores among first-year associate degree and bachelor degree nursing students in the Netherlands [16, 31]. Although physicians and nurses with postgraduate education had higher knowledge scores, all of the respondents scored below the equivalent of a D-grade in the US on a 100-point scale [31]. The finding on unsatisfactory geriatric knowledge among healthcare providers in our convenient sample was concordant with two other studies on geriatric knowledge, though they were focused on geriatric palliative care in Vietnam [42, 43].

Despite the unsatisfactory geriatric knowledge scores, respondents were highly confident about their answers. Roethler et al. [44] reported a similar discordance between the geriatric knowledge score and confidence of healthcare providers in their knowledge in the United States. This finding of high certainty suggests that healthcare providers may lack internal motivation to seek geriatric training because they are unaware of their knowledge gaps [45].

Consistent with another study in Vietnam, [42] physicians scored higher than nurses on geriatric knowledge in the pilot study. Our finding may be explained by differences in the levels of formal education between the two groups of healthcare providers, as postgraduate education was associated with higher geriatric knowledge scores in this study. The majority (59%) of nurses in our study had less than four years of formal education training, which is similar to the nursing workforce composition in Vietnam [46, 47]. It is crucial that nurses are equipped with proficient skill and knowledge in caring for geriatric patients given how influential they are in the care and vital to meeting the diverse needs of older adults [1]. Exposure to geriatric curriculum during formal education and regular contact with older adults may increase interest in acquiring knowledge about geriatric care [1, 2, 48].

In contrast to our findings, other researchers reported an association between higher level of geriatric knowledge among health professional students with geriatric training [49–51]. While the crude coefficient for geriatric training was associated with higher knowledge, the effect was only significant at *p*-values less than 0.10 in the MLR model. This discordance may be due to differences in the geriatric training content, delivery modality, and teaching strategies, as well as time elapsed since training or rates of recall among the study’s respondents [52]. We did not assess these factors in this pilot study. These measures may have been useful in identifying effective modes of education and estimating its impact on the geriatric knowledge of healthcare providers. Furthermore, contextual and structural factors that may influence the geriatric knowledge of healthcare providers were not measured in this study. It is plausible that institutional factors may facilitate or impede access to mentorship, supervision, and continuing education on geriatric care. Similarly, the translation of geriatric knowledge into appropriate care was not observed or measured in this study. Institutional factors, such as workload and team collaboration, may promote or hinder knowledge translation.

Vietnam’s publicly-funded healthcare delivery utilizes referrals and the lowest-level facilities (commune health centers) provide primary care, especially in rural and mountainous regions where the majority (72.5%) of older adults maintain residence [53, 54]. Secondary nurses are more likely to staff commune health centers [35]. Since lower-level facilities are more accessible and affordable than central facilities for older adults, our finding supports further assessment of the geriatric competency of healthcare providers at lower-level facilities.

Our pilot study suggests geriatric knowledge gaps related to the physiopathology of geriatric conditions, communication techniques with sensory impaired older adults, and differentiating age related changes from abnormal changes or symptoms. Similarly, knowledge gaps related to geriatric conditions, such as delirium and incontinence care, were reported among Turkish and Dutch nurses [55, 56]. Comprehensive geriatric knowledge is necessary for healthcare providers to provide evidence-based information on managing these conditions to older adults and their caregivers. Educational interventions, including coaching and mentoring, are viable means of improving healthcare providers’ geriatric knowledge [48, 52, 57−59].

Further, geriatric knowledge is associated with positive attitude towards older adults [60]. Future studies could include qualitative research on geriatric attitudes and learning gaps perceived by health providers in Vietnam.

These limitations should be considered when interpreting the pilot study results. First, the quota and convenience sampling of healthcare providers from Hanoi, which is mostly urban and suburban, is susceptible to selection bias. In addition, healthcare providers were not recruited from the private sector and health facilities managed by other ministries, including the military health system. Consequently, the result may not be generalizable to other healthcare providers. The inclusion of at least two health facilities for each facility level likely broadened healthcare provider selection and potentially avoided some of the selection bias. Relying on the clinical supervisors to facilitate recruitment may have compounded the bias and threatened the interval validity of the study. However, the clinical roles and experience level of the respondents were varied and their demographics were similar to the healthcare providers at all levels of the health facilities, including the geriatric specialist hospital at the central level [36, 47].

The cross-sectional design is limited because knowledge instruments may become obsolete as new evidence advances geriatric care over time. Longitudinal study designs of a nationally representative sample would yield more comprehensive information regarding the geriatric knowledge gaps of healthcare providers over time. The use of interviewers may have increased the risk of social desirability bias in the certainty construct. However, the confidentiality of responses was communicated to respondents and the MLR model did not show any significant interviewer effect.

## Conclusions

The VKOP-Q is a validated instrument to assess geriatric knowledge among healthcare providers in Vietnam. The level of geriatric knowledge among healthcare providers in the pilot study was unsatisfactory, which supports the need for further assessment of geriatric knowledge among a nationally representative sample of healthcare workers. Continuing education, professional development, supervision, and ongoing mentoring of less experienced healthcare providers, especially at lower facility levels, are learning opportunities to capacitate the current healthcare workforce with geriatric knowledge.

## Electronic supplementary material

Below is the link to the electronic supplementary material.

Appendix I. Table 1: Proportion of correct responses for each KOP-Q item (N=112)

## Data Availability

The datasets used during the current study are available from the corresponding author on reasonable request.

## Abbreviations

CVI: Content Validity Indexing
KOP-Q: Knowledge about Older Patient’s Quiz
MLR: Multiple Linear Regression
VKOP-Q: Vietnamese Knowledge about Older Patient’s Quiz
