# Supplementary material for: Evidence-based geriatric knowledge among healthcare providers in Vietnam: adaptation, validation, and pilot of the knowledge about older patients quiz
Source: BMC Geriatr. 2023 May 12;23:287. doi: 10.1186/s12877-023-03958-3 (PMC10182704; doi:10.1186/s12877-023-03958-3)
Supplement: Supplementary file 1 — Appendix I. Table 1: Proportion of correct responses for each KOP-Q item (N=112) [file 12877_2023_3958_MOESM1_ESM.docx]

**Appendix I**

**Table 1: Proportion of correct responses for each KOP-Q item (N=112)**

|  | **KOP-Q Item** | **Percent Correct** | **Percent Certainty** |
| --- | --- | --- | --- |
| 1 | Forgetfulness, concentration issues, and indecisiveness are parts of aging rather than indicators of depression. | 15 | 83 |
| 2 | Unexpected urinary incontinence in an older person may indicate that the person is suffering from a urinary tract infection. | 18 | 85 |
| 3 | Patients with a cognitive disorder, such as dementia, are at greater risk for delirium. | 71 | 77 |
| 4 | Malnutrition can have negative effects on thinking and observation skills. | 82 | 81 |
| 5 | In general, older people are more sensitive to medication because their kidney and liver functions are declining. | 76 | 82 |
| 6 | Meeting with families during patient assessment is required only for persons suffering from dementia. | 89 | 91 |
| 7 | For older people, bed rest is important to enhance recovery. | 59 | 86 |
| 8 | Patients rarely remember that they were anxious and/or restless during delirium. | 15 | 82 |
| 9 | Older people need less fluid because they exercise less. | 79 | 84 |
| 10 | Asking patients whether they have fallen in the past 6 months is a good way of assessing risk of falling. | 77 | 83 |
| 11 | Pressure that cuts off the blood supply to tissue for two hours may result in pressure ulcers. | 55 | 81 |
| 12 | Depression is recognized in older people less frequently than it is in younger people. | 60 | 78 |
| 13 | Lowering the frequency of a medication is an effective intervention to achieve (medication) adherence by patients. | 66 | 82 |
| 14 | Incontinent patients must have their soiled clothing changed but do not need to be placed on the toilet afterwards. | 56 | 81 |
| 15 | It is good to have older people drink more often, because they have a reduced thirst sensation | 17 | 80 |
| 16 | In the case of delirium, bright lighting should be used to illuminate all of the corners of the room. | 47 | 76 |
| 17 | Medication may cause geriatric problems such as memory deficits, incontinence, falling, and depression. | 69 | 78 |
| 18 | Overburdening of family caregivers may lead to abuse of the person for whom they are providing care. | 88 | 81 |
| 19 | It is good to provide extensive instruction about how to complete tasks to patients with apraxia. | 7 | 86 |
| 20 | When speaking to hearing-impaired older patients, it is best to speak at normal volume. | 39 | 84 |
| 21 | An older person with a BMI of >25 cannot be undernourished. | 54 | 86 |
| 22 | In the case of difficulty swallowing, all medicines must be ground to ensure that patients ingest them. | 34 | 87 |
| 23 | In the case of depression, memory problems may occur. | 95 | 86 |
| 24 | Most family caregivers do not need additional support from homecare services. | 88 | 85 |
| 25 | As a health provider, you have to speak clearly into the ear of hearing-impaired older patients. | 35 | 85 |
| 26 | Pain medication should be administered to older people as little as possible, due to the possibility of addiction. | 37 | 82 |
| 27 | We identify pressure ulcers only if blister formation or abrasions have occurred. | 79 | 86 |
| 28 | In the case of delirium, activities should be spread out evenly over the day. | 75 | 78 |
| 29 | The risk of falling is higher for people in the hospital setting than in those who are living at home. | 17 | 82 |
| 30 | Stress incontinence may occur in patients who are not capable of opening their own trousers. | 28 | 77 |
